# Cascara Sagrada

**Published:** 1888-08

**Authors:** 


CASCARA SAGRADA.—We invite special attention to the advertisement on last outside cover page in regard to the specific action of Cascara Sagrada in rheumatism. The disease has so long been the opprobrium medicorium of the profession, that it is well to note carefully and test any new agent that promises relief in this troublesome affection.

